# Assessment of Guideline Discordance With Antimicrobial Prophylaxis Best Practices for Common Urologic Procedures

**DOI:** 10.1001/jamanetworkopen.2018.6248

**Published:** 2018-12-21

**Authors:** Chelsea Khaw, Anthony D. Oberle, Brian C. Lund, Jason Egge, Brett H. Heintz, Bradley A. Erickson, Daniel J. Livorsi

**Affiliations:** 1 Iowa City VA Health Care System, Iowa City; 2 Department of Urology, University of Iowa Hospitals and Clinics, Iowa City; 3 Division of Infectious Diseases, University of Iowa Hospitals and Clinics, Iowa City

## Abstract

**Importance:**

The American Urological Association guidelines recommend 24 or fewer hours of antimicrobial prophylaxis for most urologic procedures. Continuing antimicrobial therapy beyond 24 hours may carry more risks than advantages.

**Objectives:**

To assess guideline discordance of antimicrobial prophylaxis for common urologic endoscopic procedures, and to identify opportunities for improving antimicrobial prescribing through future stewardship interventions.

**Design, Setting, and Participants:**

This multicenter cohort study conducted manual audits of medical records of 375 patients who underwent 1 of 3 urologic procedures (transurethral resection of bladder tumor [TURBT], transurethral resection of the prostate [TURP], and ureteroscopy [URS]) at 5 Veterans Health Administration facilities from January 1, 2016, to June 30, 2017. Antimicrobial prescribing practices across the national Veterans Health Administration system were assessed using the administrative data for 29 530 records.

**Main Outcomes and Measures:**

Guideline discordance was assessed in the medical record review. Excessive postprocedural antimicrobial use was measured in the national administrative data analysis.

**Results:**

The medical records of a total of 375 patients were manually reviewed. Among the 375 patients, 366 (97.6%) were male and 9 (2.4%) were female, with a mean (SD) age of 64.2 (10.9) years and a predominantly white race/ethnicity (289 [77.1%]). In addition, 29 530 patient records in the national administrative database were assessed. Among the patient records, 28 938 (98.0%) were male and 592 (2.0%) were female with a mean (SD) age of 69.1 (10.2) years and a predominantly white race/ethnicity (23 297 [78.9%]). Among the manually reviewed medical records, periprocedural or postprocedural antimicrobial prescribing was guideline discordant in 217 patients (57.9%). Postprocedural antimicrobial agents were continued beyond 24 hours in 211 patients (56.3%) and were guideline discordant in 177 patients (83.9%), with a median (interquartile range) duration of 3 (3-5) days of unnecessary antimicrobial therapy. In the analysis of national administrative data, excessive postprocedural antimicrobial agents were prescribed in 10 988 of 29 350 patient records (37.2%), with a median (interquartile range) of 3 (2-6) excess days. For any given facility, a statistically significant correlation was observed in the frequency of postprocedural antimicrobial prescribing between any 2 procedures, indicating that facilities with higher rates of excessive use for 1 procedure also had higher rates for another procedure: TURP and TURBT (ρ = 0.719; 95% CI, 0.603-0.803; *P* < .001), TURP and URS (ρ = 0.629; 95% CI, 0.476-0.741; *P* < .001), and TURBT and URS (ρ = 0.813; 95% CI, 0.724-0.873; *P* < .001).

**Conclusions and Relevance:**

In this study of patients who underwent common urologic procedures, the rate of guideline-discordant antimicrobial use was high mostly because of overprescribing of postprocedural antimicrobial agents; future antimicrobial stewardship interventions should target the postprocedural period.

## Key Points

**Question:**

Do urologic clinicians within the Veterans Health Administration system adhere to the American Urological Association’s antimicrobial prophylaxis guideline for endoscopic urologic procedures?

**Findings:**

In this cohort study analyzing the medical records of 375 patients at 5 Veterans Health Administration hospitals and the administrative data of 29 530 records throughout the entire Veterans Health Administration system, antimicrobial prescribing was guideline discordant in nearly 60% of patients, the rate of excessive postprocedural antimicrobial use was high, and nearly 40% of records received a median of 3 excess days of antimicrobial therapy. Agreement between these 2 data sources was high.

**Meaning:**

In patients who underwent common urologic procedures, the rates of guideline-discordant antimicrobial use were high, mainly because of overprescribing of postprocedural antimicrobial agents.

## Teaser

This cohort study evaluates the use of and prescribing patterns for antimicrobial medications in patients who underwent urologic procedures at Veterans Health Administration hospitals.

## Introduction

Antimicrobial misuse and overuse are key factors in the emergence of antimicrobial-resistant bacteria,^1^ creating a need to improve antimicrobial prescribing.^2^ Antimicrobial stewardship programs have been shown to be effective in reducing unnecessary antimicrobial prescriptions and are a requirement for accreditation by The Joint Commission.^3^

Antimicrobial use in surgical specialties is a potential target for antimicrobial stewardship efforts because certain subspecialties prescribe large quantities of antimicrobial agents. For example, urologists prescribed 6 million antimicrobial prescriptions in the United States in 2011 alone.^4^ Although urologists represented only 1.06% (9 210) of all prescribing providers in 2015, they were the eighth highest prescribers of outpatient antimicrobial agents among all specialties.^5^

Urologists commonly prescribe antimicrobial agents before, during, and after urologic procedures. The Best Practice Policy Statement on Urologic Surgery and Antimicrobial Prophylaxis, published by the American Urological Association (AUA) in 2008, recommends the administration of antimicrobial prophylaxis for 24 or fewer hours during the perioperative period.^6^ Exceptions to this recommendation apply to patients with an infection present at the time of the procedure. The AUA guidelines note that, in the absence of preexisting and untreated bacterial colonization, there is “no evidence that prophylaxis should extend beyond 24 hours following a procedure.”^6^^(p13)^

Surveys have demonstrated variability among urologists in their perceived adherence to the professional guidelines.^7,8^ Few studies, however, have assessed whether antimicrobial prescribing behavior among urologists actually follows guideline recommendations.^9,10,11^ Mossanen et al^10^ used a large health care database to assess antimicrobial appropriateness in patients who had urologic procedures from 2007 to 2012. The study found patients received antimicrobial prescriptions that were compliant with the AUA guidelines less than 60% of the time. However, these findings were based entirely on administrative data; thus, identifying unique patient care situations in which deviation from the AUA guidelines may have been justified was not possible.

In the current study, we evaluated antimicrobial use in patients who underwent 3 common urologic procedures within the Veterans Health Administration (VHA), the largest nationally integrated health care system in the United States. To avoid making assumptions about antimicrobial prescribing practices, we performed a retrospective manual medical record review of antimicrobial prescribing. Findings from this review were compared with national administrative data to validate the accuracy of the national data extraction. The primary objective was to determine the overall frequency of guideline-discordant prescribing of antimicrobial prophylaxis for 3 endoscopic urologic procedures. We hypothesized that postprocedural antimicrobial medications frequently would be prescribed for durations that were longer than necessary. Secondary objectives were to compare antimicrobial prescribing patterns across individual VHA hospitals and to describe treatment practices for asymptomatic bacteriuria detected before the procedure. Our goal was to identify opportunities to improve antimicrobial prophylaxis prescribing through future antimicrobial stewardship interventions.

## Methods

The research protocol was approved by the University of Iowa Institutional Review Board and the Iowa City Veterans Affairs Administration Research and Development Committee. Patient consent was waived by the University of Iowa Institutional Review Board. This study followed the Strengthening the Reporting of Observational Studies in Epidemiology (STROBE) reporting guideline.

### Manual Medical Record Review

We conducted a multicenter retrospective cohort study at 5 unique VHA sites. Patients at these sites were eligible if they underwent a medical procedure with a *Current Procedural Terminology* code or an *International Statistical Classification of Diseases and Related Health Problems, Tenth Revision (ICD-10), Procedure Coding System* code for transurethral resection of the prostate (TURP), transurethral resection of bladder tumor (TURBT), or ureteroscopy (URS) between January 1, 2016, and June 30, 2017 (eTable 1 in the Supplement). These 3 procedures were selected because they were all common procedures performed endoscopically with no skin incision required. Patients with multiple procedures were restricted to the first observed procedure within the study period.

All VHA hospitals were eligible for selection if at least 20 of each procedure (TURP, TURBT, and URS) were performed therein during the study period. This criterion eliminated sites with low volumes, which may have limited their expertise with these procedures. The sites were then divided evenly into quintiles, on the basis of the number of the 3 procedures performed within the observation period. To create a cohort with varying levels of experience with these procedures, we randomly selected 1 site from each quintile with the exception that our own center (Iowa City VA Health Care System) was included in its quintile. We purposefully selected our site to ensure that the study findings could inform future quality improvement efforts at the center.

We included 25 randomly selected patients per procedure per site. For each patient, all clinical information during the periprocedural period was obtained from the electronic medical record through the Compensation and Pension Record Interchange system. These data were reviewed by a pharmacy resident (C.K.) and urology resident (A.D.O.) on our team. Urine culture results from 90 days before the procedure were reviewed to detect potential colonization. If the urine culture grew no microorganisms or only grew common skin commensals, the sample result was considered to be negative.

For each patient, antimicrobial use was reviewed during the preprocedural, periprocedural, and postprocedural periods. *Preprocedural antimicrobial* was defined as any antimicrobial agent prescribed by a urologic practitioner for the treatment of asymptomatic or symptomatic bacteriuria and candiduria during the 90 days before the procedure. Preprocedural antimicrobial use for bacteriuria and candiduria was considered appropriate if (1) the prescribed antimicrobial agent had activity against the microorganisms isolated from the patient’s urine culture and (2) the duration of the antimicrobial agent was 5 to 7 days (±2 days).^12^ Longer antimicrobial courses were considered appropriate if chronic infection (eg, an infected and retained stone) was a concern. Antimicrobial agents prescribed by nonurologic practitioners were not included in the analysis. *Periprocedural antimicrobial* was defined as a systemic antimicrobial agent administered 1 to 2 hours before and/or during a qualifying urologic procedure. *Postprocedural antimicrobial* was defined as any antimicrobial agent prescribed by a urologist after the procedure was completed.

Guideline concordance or discordance was assessed during the periprocedural and postprocedural periods. *Guideline concordance* was defined as compliance with the 2008 AUA guidelines and was determined by 2 criteria. The first criterion (periprocedural) was the selection of an AUA-recommended antimicrobial prophylaxis agent without known resistance to that agent or selection of a non-AUA prophylaxis agent with an appropriate indication (eTable 2 in the Supplement), such as a documented allergy or known resistance to all AUA-recommended agents. The second criterion (postprocedural) was either the discontinuation of an antimicrobial agent within 24 hours of the procedure’s completion or the documentation of an appropriate clinical indication or apparent need (based on medical record review) for continuing the antimicrobial agent for longer than 24 hours. Postprocedural antimicrobial agents prescribed for more than 24 hours were considered guideline concordant for symptomatic bacteriuria, nonurinary infections, and prophylaxis after iatrogenic perforation of the urinary tract. For patients with a history of urinary stones, prolonged postprocedural antimicrobial agents were considered guideline concordant if stone fragments were retained after the procedure and these stone fragments were thought to be infected according to either microbiologic data or clinical findings. Because preprocedural urine cultures may not have been accurate (eg, in patients with obstructing lesions), urine cultures collected during the procedure were also used to determine guideline concordance in the postprocedural period.

The medical records of all patients who received either a non–AUA-recommended antimicrobial or postprocedural antimicrobial agent beyond 24 hours were reviewed independently by the infectious disease physician (D.J.L.) and urology resident (A.D.O.) on our team to determine whether the indication was guideline concordant. We resolved by consensus any differences in opinion among us, and we assessed guideline concordance or discordance for all patients.

### National Data Extraction

We obtained national administrative data for all VHA medical centers from the VHA Corporate Data Warehouse, and we accessed these data through the VHA Informatics and Computing Infrastructure. All TURBT, TURP, and URS procedures performed from January 1, 2016, to June 30, 2017 (the same observation period used in the medical record review), were identified from inpatient and outpatient encounter data using *Current Procedural Terminology* or *ICD-10, Procedure Coding System* codes (eTable 1 in the Supplement). Antimicrobial agents potentially used for a urinary tract–related indication (eTable 2 in the Supplement) were ascertained from inpatient and outpatient prescriptions dispensed within 14 days after the procedure. *Excessive postprocedural antimicrobial use* was defined as a prescription for 1 of these agents on postprocedural day 2 if the prophylaxis was part of a continuous course from the time of the procedure. This definition was used to minimize the chance of including patients who received antimicrobial agents for nonurologic or nonprophylactic indications.

### Statistical Analysis

Descriptive statistics were used to characterize the frequency of guideline-discordant antimicrobial prescribing. A κ coefficient was calculated to compare the electronic identification of antimicrobial agents prescribed on postoperative day 2 with guideline concordance as determined from manual medical record review. This comparison was performed to determine the accuracy of the national data extraction in identifying excessive postprocedural antimicrobial use.

After data were extracted from all VHA sites, those sites in which at least 20 of each procedure were performed during the study period were analyzed to determine if facility-level associations existed between the rate of excessive antimicrobial prescribing and the individual procedures. Spearman rank correlations were used to assess facility-level rates of excessive postprocedural antimicrobial prescribing and procedure counts. All analyses were performed using SAS Enterprise Guide, version 7.1 (SAS Institute Inc), and all statistical tests were unpaired and 2-tailed at the 2-sided *P* = .05 level of significance.

## Results

The medical records of 375 patients were manually reviewed. Among the 375 patients, 366 (97.6%) were male and 9 (2.4%) were female, with a mean (SD) age of 64.2 (10.9) years and a predominantly white race/ethnicity (289 [77.1%]). In addition, 29 530 patient records in the national administrative database were assessed. Among the patient records, 28 938 (98.0%) were male and 592 (2.0%) were female with a mean (SD) age of 69.1 (10.2) years and a predominantly white race/ethnicity (23 297 [78.9%]).

### Manual Medical Record Review at VHA Sites

The 5 VHA sites that were selected for inclusion in the manual medical record review were geographically diverse, including at least 1 site in the Northeast, Southeast, Midwest, and Western regions of the United States. A total of 444 patients were screened, of whom 69 (15.5%) were excluded because they did not undergo the coded procedure, leaving 375 patients (who represented 1.3% of the national cohort).

Preprocedural urine cultures were collected in 325 of 375 patients (86.7%) (Table 1). The most recent urine culture was performed a median (interquartile [IQR]) of 11 (7-19) days before the procedure, and 106 of 325 patients (32.6%) had bacteriuria or candiduria. Management of preprocedural urine culture screening and treatment was considered inappropriate in 104 patients (27.7%). The most common reason for this inappropriate classification was failure to collect a urine culture, which occurred in 49 of 104 patients (47.1%); additional reasons for inappropriateness can be found in Table 1. Forty-two of the 98 patients (42.9%) who were given a preprocedural antimicrobial prescription for bacteriuria or candiduria received the medication more than 7 days before the procedure.

**Table 1.** Preprocedural Evaluation and Treatment for Bacteriuria

Practice Under Evaluation	No. (%)			
	TURBT (n = 125)	TURP (n = 125)	URS (n = 125)	All Procedures (N = 375)
Urine culture within 90 d before the procedure				
No. collected	103 (82.4)	113 (90.4)	109 (87.2)	325 (86.7)
Days before the procedure of culture collection, median (IQR)	13 (7-22)	11 (7-17)	9 (5-18)	11 (7-19)
Frequency of positive urine culture, No./total No. (%)	22/103 (21.4)	44/113 (38.9)	40/109 (36.7)	106/325 (32.6)
Preprocedural assessment and management of bacteriuria				
Appropriate care scenarios	96 (76.8)	89 (71.2)	86 (68.8)	271 (72.3)
No positive culture; no antimicrobial prescribed	77 (61.6)	59 (47.2)	60 (48.0)	196 (52.3)
Positive culture, repeated negative results; no antimicrobial prescribed	4 (3.2)	0	2 (1.6)	6 (1.6)
Positive culture; appropriate antimicrobial prescribed	15 (12.0)	30 (24.0)	24 (19.2)	69 (18.4)
Inappropriate care scenarios	29 (23.2)	36 (28.8)	39 (31.2)	104 (27.7)
No preprocedure culture ordered	22 (17.6)	12 (9.6)	16 (12.8)	50 (13.3)
Positive culture; inappropriate antimicrobial prescribed	0	1 (0.8)	0	1 (0.3)
Positive culture; no antimicrobial observed	3 (2.4)	11 (8.8)	12 (9.6)	26 (6.9)
Culture-negative results; antimicrobial prescribed	4 (3.2)	12 (9.6)	11 (8.8)	27 (7.2)
Timing of antimicrobial initiation				
No. of patients	19	43	35	97
Within 3 d before the procedure	1 (5.3)	7 (16.3)	7 (20.0)	15 (15.5)
Within 4-7 d before the procedure	5 (26.3)	23 (53.5)	12 (34.3)	40 (41.2)
More than 7 d before the procedure	13 (68.4)	13 (30.2)	16 (45.7)	42 (43.3)

Abbreviations: IQR, interquartile range; TURBT, transurethral resection of bladder tumor; TURP, transurethral resection of the prostate; URS, ureteroscopy.

Periprocedural antimicrobial prescribing was guideline discordant in 66 of 375 patients (17.6%), and postprocedural prescribing was guideline discordant in 182 (48.5%) patients (Table 2). In total, 217 of 375 patients (57.9%) received guideline-discordant prescriptions during either the periprocedural or postprocedural period. Postprocedural antimicrobial agents were continued beyond 24 hours in 211 of 375 patients (56.3%). An appropriate clinical indication for continuation was documented in 34 patients (16.1%), with a median (IQR) duration of 7 (4-11) days. Postprocedural continuation of antimicrobial prophylaxis was guideline discordant among the remaining 177 (83.9%) records, with a median (IQR) duration of 3 (3-5) excess days of therapy.

**Table 2.** Assessment of Periprocedural and Postprocedural Antimicrobial Appropriateness

Practice Under Evaluation	No. (%)			
	TURBT (n = 125)	TURP (n = 125)	URS (n = 125)	All Procedures (N = 375)
Periprocedural period^a^				
Guideline-concordant antimicrobial prescribed^b^	113 (90.4)	107 (85.6)	89 (71.2)	309 (82.4)
Guideline-discordant therapy	12 (9.6)	18 (14.4)	36 (28.8)	66 (17.6)
Guideline-discordant antimicrobial prescribed	1 (0.8)	10 (8.0)	26 (20.8)	37 (9.9)
No documentation of antimicrobial prescribed	11 (8.8)	8 (6.4)	10 (8.0)	29 (7.7)
Postprocedural period				
Guideline-concordant therapy^c^	71 (56.8)	61 (48.8)	61 (48.8)	193 (51.5)
No antimicrobial prescribed	65 (52.0)	50 (40.0)	44 (35.2)	159 (42.4)
Antimicrobial for appropriate indication	6 (4.8)	11 (8.8)	17 (13.6)	34 (9.1)
Duration of therapy, median (IQR), d	2 (1-8)	11 (4-16)	4 (4-9)	7 (4-11)
Guideline-discordant therapy	54 (43.2)	64 (51.2)	64 (51.2)	182 (48.5)
Unable to evaluate therapy	1 (0.8)	1 (0.8)	3 (2.4)	5 (1.3)
Antimicrobials prescribed, inappropriate indication	53 (42.4)	63 (50.4)	61 (48.8)	177 (47.2)
Duration of excess therapy, median (IQR), d	3 (3-5)	3 (3-5)	3 (3-5)	3 (3-5)
Overall guideline concordance, both periprocedural and postprocedural periods				
Concordant therapy in both periods	59 (47.2)	52 (41.6)	47 (37.6)	158 (42.1)
Discordant therapy in either period	66 (52.8)	73 (58.4)	78 (62.4)	217 (57.9)

Abbreviations: IQR, interquartile range; TURBT, transurethral resection of bladder tumor; TURP, transurethral resection of the prostate; URS, ureteroscopy.

^a^ Periprocedural period is 1 to 2 hours before and/or during a qualifying urologic procedure.

^b^ Guideline-concordant periprocedural therapy was defined as the selection of an American Urological Association–recommended antimicrobial agent without known resistance to that agent or selection of a non–American Urological Association agent with an appropriate indication.

^c^ Guideline-concordant postprocedural therapy was defined as the discontinuation of an antimicrobial agent within 24 hours of the procedure’s completion or the documentation of an appropriate clinical indication or apparent need (based on medical record review) for continuing the antimicrobial agent for longer than 24 hours.

Among the 5 VHA sites, rates of guideline-discordant antimicrobial prescribing varied (Table 3). The frequency of guideline discordance in either period ranged from 13 (17.3%) to 66 (88.0%) across the 5 sites.

**Table 3.** Facility Variation in Guideline-Discordant Antimicrobial Prescribing in the Periprocedural and Postprocedural Periods

Variable	Site^a^					Total
	1	2	3	4	5	
Total procedure volume, No.	212	323	332	480	573	NA
Medical records reviewed, No.	75	75	75	75	75	375
Guideline-discordant prescribing, No. (%)						
Periprocedural period^b^	3 (4.3)	29 (38.7)	7 (9.3)	11 (14.7)	16 (21.3)	66 (17.6)
Postprocedural period^c^	63 (84.0)	33 (44.0)	19 (25.3)	3 (4.0)	64 (85.3)	182 (48.5)
Either period	64 (85.3)	49 (65.3)	25 (33.3)	13 (17.3)	66 (88.0)	217 (57.9)

Abbreviation: NA, not applicable.

^a^ One site was selected from each quintile of total procedure volume, with 75 patients randomly selected for review from each site.

^b^ Periprocedural period is 1 to 2 hours before and/or during a qualifying urologic procedure.

^c^ Postprocedural period is any time after the procedure was completed.

### National Administrative Data

In our analysis of the national administrative data, 29 530 procedures were identified during the observation period. Excessive postprocedural antimicrobial agents were prescribed in 10 988 (37.2%) of 29 350 patient records with a median (IQR) of 3 (2-6) days of excess therapy (Table 4). Excessive postprocedural antimicrobial use varied across procedures: 3234 (40.3%) for TURP, 4894 (32.9%) for TURBT, and 2860 (43.0%) for URS. Agreement was found between the administrative data on antimicrobial agents prescribed on postoperative day 2 and guideline discordance determined by manual medical record reviews (κ = 0.82; 95% CI, 0.76-0.88).

**Table 4.** Frequency of Excessive Postprocedural Antimicrobial Prescribing

Variable	Procedure			Total
	TURBT	TURP	URS	
Procedural count, No.	14 858	8027	6645	29 530
Excessive postprocedural antimicrobial prescribing, No. (%)^a^	4894 (32.9)	3234 (40.3)	2860 (43.0)	10 988 (37.2)
Days of excess therapy, median (IQR)^b^	3 (2-5)	3 (2-6)	4 (2-6)	3 (2-6)

Abbreviations: IQR, interquartile range; TURBT, transurethral resection of bladder tumor; TURP, transurethral resection of the prostate; URS, ureteroscopy.

^a^ Continuation of inpatient antimicrobial therapy for more than 2 days after the procedure, or an outpatient antimicrobial agent dispensed with 2 or more days’ supply on the day of or day after the procedure, or an outpatient antimicrobial agent dispensed on the second day after the procedure as a continuation of previous inpatient antimicrobial use.

^b^ Days of therapy beyond the recommended duration among patients who received excessive postprocedural antimicrobial agents.

Wide variation in excessive postprocedural antimicrobial use was found at the facility level (Table 5). Facilities with higher procedural counts for TURP were less likely to prescribe excessive postprocedural antimicrobial prophylaxis (ρ = –0.387; 95% CI, –0.545 to –0.198; *P* < .001). A similar correlation was not seen for TURBT (ρ = –0.133; 95% CI, –0.313 to 0.057; *P* = .17) or URS (ρ = –0.017; 95% CI, –0.226 to 0.194; *P* = .88). The frequency of excessive postprocedural antimicrobial use within a given VHA facility had statistically significant positive correlations across procedures, indicating that facilities with higher rates of excessive use for 1 procedure also had higher rates for another procedure: TURP and TURBT (ρ = 0.719; 95% CI, 0.603-0.803; *P* < .001), TURP and URS (ρ = 0.629; 95% CI, 0.476-0.741; *P* < .001), and TURBT and URS (ρ = 0.813; 95% CI, 0.724-0.873; *P* < .001).

**Table 5.** Variation in Excessive Postprocedural Antimicrobial Prescribing

Variable	Procedure		
	TURBT	TURP	URS
Medical centers, No.	104	94	87
Procedural count, mean (SD), No.	134 (78)	84 (50)	74 (47)
Frequency of excessive postprocedural antimicrobial prescribing, median (IQR), %^a^^,^^b^	27.9 (14.7-55.0)	43.5 (26.4-62.3)	39.1 (24.4-58.1)

Abbreviations: IQR, interquartile range; TURBT, transurethral resection of bladder tumor; TURP, transurethral resection of the prostate; URS, ureteroscopy.

^a^ Continuation of inpatient antimicrobial therapy for more than 2 days after the procedure, or an outpatient antimicrobial agent dispensed with 2 or more days’ supply on the day of or day after the procedure, or an outpatient antimicrobial agent dispensed on the second day after the procedure as a continuation of previous inpatient antimicrobial use.

^b^ Medians and IQRs reflect a ranking of Veterans Health Administration sites according to the frequency of excessive postprocedural antimicrobial use.

## Discussion

The 2008 AUA guidelines recommend stopping antimicrobial agent use within 24 hours of endoscopic procedures. This recommendation is based on several randomized prospective studies of a variety of urologic procedures that found no difference in postprocedural outcomes in patients who received 24 or fewer hours of antimicrobial prophylaxis compared with patients who received more extended antimicrobial courses.^13,14,15,16,17,18,19,20^

However, our findings indicate that postprocedural antimicrobial agents were commonly prescribed by urology services across the VHA for durations that well exceeded 24 hours. Our analysis of administrative data indicated that 37.2% of patients received excessive postprocedural antimicrobial agents for a median duration of 3 days. This number does not necessarily reflect the number of patients who received guideline-discordant antimicrobial prophylaxis, but the manual medical record review we conducted would suggest that only 16.1% of these patients likely had an appropriate indication for extended therapy.

Other studies have found that excessive postprocedural antimicrobial agents are commonly prescribed after urologic procedures. For example, Calvert et al^9^ reviewed 2007 to 2012 data from a collaborative health care network of more than 2900 US hospitals and found that 20% to 35% of patients who underwent urologic procedures for genitourinary cancers received extended antimicrobial prophylaxis. None of these patients underwent the 3 procedures reviewed in the current study. Mossanen et al^10^ reviewed data from the same 2007 to 2012 database used by Calvert et al^9^ and found that the mean duration of antimicrobial prophylaxis exceeded 24 hours for multiple urologic procedures but not for TURP, TURBT, or URS. Unlike the current study, Mossanen et al^10^ excluded a substantial proportion of patients because the prescribed antimicrobial class was changed within 24 hours of the procedure and thus may have underestimated the rate of guideline-discordant postprocedural antimicrobial use.

Excessive postprocedural antimicrobial use varied across the more than 80 VHA sites included in our national analysis. Interfacility variation in excessive postprocedural antimicrobial prescribing ranged from 14.7% to 62.3% (Table 3). Such a high degree of interfacility variability may reflect a lack of guideline awareness, patient-specific factors that vary across sites, and skepticism about guideline recommendations among certain prescribers.^21,22,23^ The interfacility variability may also reflect differences in the process of prescribing across sites. For example, some facilities may rely more heavily on physicians-in-training or automated protocols to prescribe antimicrobial prophylaxis, which could affect the frequency of guideline concordance. Additional studies are needed to determine the factors that drive guideline-discordant prescribing among urologists, including any site-specific factors that explain the high degree of interfacility variability we observed.

This study found that preprocedural screening and treatment for bacteriuria were largely appropriate, although both were frequently performed well before the procedure. The AUA guidelines are not clear on when to screen and treat for asymptomatic bacteriuria. Studies have shown that high-risk patients with baseline asymptomatic bacteriuria, such as patients with bladder cancer, have undergone outpatient urologic procedures with either no or single-dose antimicrobial prophylaxis without increasing the risk for adverse outcomes.^24,25,26^ These findings would seem to support the recommendations from the Infectious Diseases Society of America: antimicrobial treatment for asymptomatic bacteriuria should be initiated immediately before the procedure and stopped immediately after the procedure.^27^

The present study indicates that the postprocedural period should be the primary target for antimicrobial stewardship interventions. Reducing unnecessary postprocedural antimicrobial use may help decrease the rates of antimicrobial resistance, the incidence of *Clostridioides difficile*, and other adverse patient outcomes.^2^ A study using a large national database of urologic surgical procedures performed for genitourinary cancers found that excessive postprocedural antimicrobial prophylaxis was associated with an increased risk for *C difficile* infection.^9^

Previous studies have described strategies that can help improve antimicrobial prescribing for urologic procedures. In a Chinese hospital, a pharmacist-driven intervention with real-time feedback achieved reductions in antimicrobial use and associated costs among patients who had urologic procedures.^28^ At an Italian hospital, several positive outcomes were seen after the implementation of a new surgical prophylaxis protocol based on the 2010 recommendations of the European Association of Urology.^29,30^ In the period after the implementation of this protocol, the consumption of fluoroquinolones and aminoglycosides substantially decreased, whereas the use of trimethoprim-sulfamethoxazole substantially increased. In addition, the incidence of symptomatic postoperative infections was unchanged and the prevalence of antimicrobial resistance among certain uropathogens was reduced.^30^ These studies show that pharmacist-driven and protocol-based interventions can be effective in reducing unnecessary antimicrobial use among patients who underwent urologic procedures.

### Strengths and Limitations

The strengths of this study include the use of medical record review to assess guideline concordance. Because we extracted the data by manually reviewing medical records, we were able to identify situations in which deviation from AUA guidelines was appropriate. In contrast, the largest studies on this topic, both of which relied exclusively on the same administrative database, made several assumptions about what did and did not qualify as appropriate antimicrobial use.^9,10^ Furthermore, the large sample size from the national data analysis was validated by medical record review. Overall, there was excellent agreement between these 2 data sources.

This study has limitations as well. First, we may have underestimated unnecessary postprocedural antimicrobial use, as new guidelines recommend the discontinuation of use after the incision has been closed.^31,32,33^ Second, we were unable to account for any additional antimicrobial prescriptions outside the VHA. Third, the definition of guideline-discordant postprocedural antimicrobial use in our manual review was more restrictive than the definition of excessive postprocedural antimicrobials in our national data analysis. These discrepancies led to an underestimation of excessive postprocedural antimicrobial use in the national data analysis. Fourth, a small proportion of procedures identified in the national data may have been miscoded. In our manual review, approximately 16% of patients were excluded for this reason. However, because these excluded patients likely did not require more than 24 hours of postprocedural antimicrobial agents, such a misclassification should not have substantially affected our overall findings. Fifth, we cannot comment on how the process of antimicrobial prescribing varied across sites, but this does not change the substantial opportunity for improvement that this study has identified. Sixth, some clinical situations may necessitate the continuation of antimicrobial use beyond the recommended time frame indicated by the AUA guidelines. Documentation of these clinical situations and the surgeon’s reason for deviating from guidelines may have been lacking. Seventh, it is unclear how well these findings apply to non-VHA hospitals, but note that many urologists are trained at academic medical centers that include a VHA hospital. How urologists learn to prescribe antimicrobial prophylaxis during residency training may affect their future prescribing patterns in independent practice.

## Conclusions

In patients undergoing common urologic procedures, the rate of guideline-discordant antimicrobial use was high, mostly because of overprescribing of postprocedural antimicrobial prophylaxis. Future antimicrobial stewardship interventions should target the postprocedural period with the goal of reducing unnecessary antimicrobial use.

## Notes

Supplement. **eTable 1.** CPT and ICD-10 PCS Codes Utilized in the Administrative Data Analysis **eTable 2.** Recommended Antimicrobial Prophylaxis for Urologic Procedures According to American Urological Association Guidelines

## References

[1] Llor C, Bjerrum L Antimicrobial resistance: risk associated with antibiotic overuse and initiatives to reduce the problem. Ther Adv Drug Saf. 2014;5(6):-. doi:10.1177/2042098614554919 25436105 PMC4232501

[2] Centers for Disease Control and Prevention Antibiotic Resistance Threats in the United States. Atlanta, GA: Department of Healthcare Quality Promotion; 2013.

[3] Joint Commission on Hospital Accreditation Approved: new antimicrobial stewardship standard. Jt Comm Perspect. 2016;36(7):e186248. 27548932

[4] Hicks LA, Bartoces MG, Roberts RM, US outpatient antibiotic prescribing variation according to geography, patient population, and provider specialty in 2011. Clin Infect Dis. 2015;60(9):1308-1316. 25747410 10.1093/cid/civ076

[5] Durkin MJ, Hsueh K, Sallah YH, ; Centers for Disease Control and Prevention Epicenters An evaluation of dental antibiotic prescribing practices in the United States. J Am Dent Assoc. 2017;148(12):878-886. doi:10.1016/j.adaj.2017.07.019 28941554 PMC5705569

[6] Wolf JS Jr, Bennett CJ, Dmochowski RR, Hollenbeck BK, Pearle MS, Schaeffer AJ; Urologic Surgery Antimicrobial Prophylaxis Best Practice Policy Panel Best practice policy statement on urologic surgery antimicrobial prophylaxis. J Urol. 2008;179(4):1379-1390. doi:10.1016/j.juro.2008.01.068 18280509

[7] Çek M, Tandoğdu Z, Naber K, ; Global Prevalence Study of Infections in Urology Investigators Antibiotic prophylaxis in urology departments, 2005-2010. Eur Urol. 2013;63(2):386-394. doi:10.1016/j.eururo.2012.09.038 23031676

[8] Bausch K, Roth JA, Seifert HH, Widmer AF Overuse of antimicrobial prophylaxis in low-risk patients undergoing transurethral resection of the prostate. Swiss Med Wkly. 2018;148:w14594. 29473939 10.4414/smw.2018.14594

[9] Calvert JK, Holt SK, Mossanen M, Use and outcomes of extended antibiotic prophylaxis in urological cancer surgery. J Urol. 2014;192(2):425-429. doi:10.1016/j.juro.2014.02.096 24603103

[10] Mossanen M, Calvert JK, Holt SK, Overuse of antimicrobial prophylaxis in community practice urology. J Urol. 2015;193(2):543-547. doi:10.1016/j.juro.2014.08.107 25196654 PMC4846360

[11] Lawson KA, Rudzinski JK, Vicas I, Carlson KV Assessment of antibiotic prophylaxis prescribing patterns for TURP: a need for Canadian guidelines? Can Urol Assoc J. 2013;7(7-8):E530-E536. doi:10.5489/cuaj.205 24032065 PMC3758947

[12] Grabe MJ, Lundström KJ Tailored perioperative antimicrobial prophylaxis in urological surgery: myth or reality? Curr Opin Urol. 2017;27(2):112-119. doi:10.1097/MOU.0000000000000363 27861259

[13] Briffaux R, Coloby P, Bruyere F, One preoperative dose randomized against 3-day antibiotic prophylaxis for transrectal ultrasonography-guided prostate biopsy. BJU Int. 2009;103(8):1069-1073. doi:10.1111/j.1464-410X.2008.08128.x 19021604

[14] Cam K, Kayikci A, Akman Y, Erol A Prospective assessment of the efficacy of single dose versus traditional 3-day antimicrobial prophylaxis in 12-core transrectal prostate biopsy. Int J Urol. 2008;15(11):997-1001. doi:10.1111/j.1442-2042.2008.02147.x 18721198

[15] Hall JC, Christiansen KJ, England P, Antibiotic prophylaxis for patients undergoing transurethral resection of the prostate. Urology. 1996;47(6):852-856. doi:10.1016/S0090-4295(96)00066-0 8677576

[16] Hara N, Kitamura Y, Saito T, Komatsubara S, Nishiyama T, Takahashi K Perioperative antibiotics in radical cystectomy with ileal conduit urinary diversion: efficacy and risk of antimicrobial prophylaxis on the operation day alone. Int J Urol. 2008;15(6):511-515. doi:10.1111/j.1442-2042.2008.02050.x 18422576

[17] Sakura M, Kawakami S, Yoshida S, Masuda H, Kobayashi T, Kihara K Prospective comparative study of single dose versus 3-day administration of antimicrobial prophylaxis in minimum incision endoscopic radical prostatectomy. Int J Urol. 2008;15(4):328-331. doi:10.1111/j.1442-2042.2008.02001.x 18380822

[18] Schaeffer AJ, Montorsi F, Scattoni V, Comparison of a 3-day with a 1-day regimen of an extended-release formulation of ciprofloxacin as antimicrobial prophylaxis for patients undergoing transrectal needle biopsy of the prostate. BJU Int. 2007;100(1):51-57. doi:10.1111/j.1464-410X.2007.06848.x 17552953

[19] Takeyama K, Takahashi S, Maeda T, Comparison of 1-day, 2-day, and 3-day administration of antimicrobial prophylaxis in radical prostatectomy. J Infect Chemother. 2007;13(5):320-323. doi:10.1007/s10156-007-0540-9 17982721

[20] Terai A, Ichioka K, Kohei N, Ueda N, Utsunomiya N, Inoue K Antibiotic prophylaxis in radical prostatectomy: 1-day versus 4-day treatments. Int J Urol. 2006;13(12):1488-1493. doi:10.1111/j.1442-2042.2006.01597.x 17118023

[21] Livorsi D, Comer AR, Matthias MS, Perencevich EN, Bair MJ Barriers to guideline-concordant antibiotic use among inpatient physicians: a case vignette qualitative study. J Hosp Med. 2016;11(3):174-180. doi:10.1002/jhm.2495 26443327 PMC4779411

[22] Cortoos PJ, De Witte K, Peetermans WE, Simoens S, Laekeman G Opposing expectations and suboptimal use of a local antibiotic hospital guideline: a qualitative study. J Antimicrob Chemother. 2008;62(1):189-195. doi:10.1093/jac/dkn143 18397925

[23] Charani E, Castro-Sanchez E, Sevdalis N, Understanding the determinants of antimicrobial prescribing within hospitals: the role of “prescribing etiquette.” Clin Infect Dis. 2013;57(2):188-196. doi:10.1093/cid/cit212 23572483 PMC3689346

[24] Aron M, Rajeev TP, Gupta NP Antibiotic prophylaxis for transrectal needle biopsy of the prostate: a randomized controlled study. BJU Int. 2000;85(6):682-685. doi:10.1046/j.1464-410x.2000.00576.x 10759665

[25] Sabbagh R, McCormack M, Péloquin F, A prospective randomized trial of 1-day versus 3-day antibiotic prophylaxis for transrectal ultrasound guided prostate biopsy. Can J Urol. 2004;11(2):2216-2219. 15182413

[26] Shigemura K, Tanaka K, Yasuda M, Efficacy of 1-day prophylaxis medication with fluoroquinolone for prostate biopsy. World J Urol. 2005;23(5):356-360. doi:10.1007/s00345-005-0024-4 16254727

[27] Nicolle LE, Bradley S, Colgan R, Rice JC, Schaeffer A, Hooton TM; Infectious Diseases Society of America; American Society of Nephrology; American Geriatric Society Infectious Diseases Society of America guidelines for the diagnosis and treatment of asymptomatic bacteriuria in adults. Clin Infect Dis. 2005;40(5):643-654. doi:10.1086/427507 15714408

[28] Zhou Y, Ma LY, Zhao X, Tian SH, Sun LY, Cui YM Impact of pharmacist intervention on antibiotic use and prophylactic antibiotic use in urology clean operations. J Clin Pharm Ther. 2015;40(4):404-408. doi:10.1111/jcpt.12275 25913640

[29] Grabe M, Bjerklund Johansen T, Botto H, EAU guidelines on urological infections 2010. https://uroweb.org/wp-content/uploads/Urological-Infections-2010.pdf. Accessed November 15, 2018.

[30] Cai T, Verze P, Brugnolli A, Adherence to European Association of Urology guidelines on prophylactic antibiotics: an important step in antimicrobial stewardship. Eur Urol. 2016;69(2):276-283. doi:10.1016/j.eururo.2015.05.010 26001610

[31] Ban KA, Minei JP, Laronga C, American College of Surgeons and Surgical Infection Society: surgical site infection guidelines, 2016 update. J Am Coll Surg. 2017;224(1):59-74. doi:10.1016/j.jamcollsurg.2016.10.029 27915053

[32] Berríos-Torres SI, Umscheid CA, Bratzler DW, ; Healthcare Infection Control Practices Advisory Committee Centers for Disease Control and Prevention guideline for the prevention of surgical site infection, 2017. JAMA Surg. 2017;152(8):784-791. doi:10.1001/jamasurg.2017.0904 28467526

[33] Allegranzi B, Zayed B, Bischoff P, ; WHO Guidelines Development Group New WHO recommendations on intraoperative and postoperative measures for surgical site infection prevention: an evidence-based global perspective. Lancet Infect Dis. 2016;16(12):e288-e303. doi:10.1016/S1473-3099(16)30402-9 27816414

