# Supplementary material for: Assessment of Guideline Discordance With Antimicrobial Prophylaxis Best Practices for Common Urologic Procedures
Source: JAMA Netw Open. 2018 Dec 21;1(8):e186248. doi: 10.1001/jamanetworkopen.2018.6248 (PMC6324350; doi:10.1001/jamanetworkopen.2018.6248)
Supplement: Supplement. — eTable 1. CPT and ICD-10 PCS Codes Utilized in the Administrative Data Analysis eTable 2. Recommended Antimicrobial Prophylaxis for Urologic Procedures According to American Urological Association Guidelines [file jamanetwopen-1-e186248-s001.pdf]

Supplementary Online Content

Khaw C, Oberle AD, Lund BC, et al. Assessment of guideline discordance with antimicrobial prophylaxis best practices for common urologic procedures. *JAMA Netw Open.* 2018;1(8):e186248. doi:10.1001/jamanetworkopen.2018.6248

eTable 1. CPT and ICD-10 PCS Codes Utilized in the Administrative Data Analysis

eTable 2. Recommended Antimicrobial Prophylaxis for Urologic Procedures According to American Urological Association Guidelines

This supplementary material has been provided by the authors to give readers additional information about their work.

©2018 Khaw C et al. *JAMA Network Open.*

eTable 1. CPT and ICD-10 PCS Codes Utilized in the Administrative Data Analysis

Procedure	CPT codes	ICD-10 PCS codes
TURBT	52224, 52234, 52235, 52240	0T5B7ZZ, 0T5B8ZZ, 0T5C7ZZ, 0T5C8ZZ, 0TBB7ZX, 0TBB7ZZ, 0TBB8ZX, 0TBB8ZZ, 0TBC7ZX, 0TBC7ZZ, 0TBC8ZX, 0TBC8ZZ
TURP	52601, 52450, 52630	0VB03ZX, 0VB03ZZ, 0VB04ZX, 0VB04ZZ, 0VB07ZX, 0VB07ZZ, 0VB08ZX, 0VB08ZZ, 0VT04ZZ, 0VT07ZZ, 0VT08ZZ, 0V503ZZ, 0V504ZZ, 0V507ZZ, 0V508ZZ
URS[1]	52343, 52344, 52345, 52346, 52351, 52352, 52353, 52354, 52355	0TC37ZZ, 0TC38ZZ, 0TC47ZZ, 0TC48ZZ, 0TC67ZZ, 0TC68ZZ, 0TC77ZZ, 0TC78ZZ, OTJ93ZZ, 0TJ97ZZ, 0TJ94ZZ, 0TJ98ZZ

TURBT = transurethral resection of bladder tumor; TURP = transurethral resection of the prostate; URS = ureteroscopy

[1] Ureteroscopies with and without urinary stones were included.

©2018 Khaw C et al. *JAMA Network Open.*

eTable 2. Recommended Antimicrobial Prophylaxis for Urologic Procedures According to American Urological Association Guidelines

Procedure	Prophylaxis indicated	Antimicrobials of choice	Alternative antimicrobials[1]	Duration of therapy
TURBT, TURP, URS	All patients	Fluoroquinolone, Sulfamethoxazole-trimethoprim	Aminoglycoside +/- ampicillin, 1st/2nd generation cephalosporin, Amoxicillin/clavulanate, Aztreonam +/- ampicillin	≤ 24 hours

TURBT = transurethral resection of bladder tumor; TURP = transurethral resection of the prostate; URS = ureteroscopy

[1] Certain antimicrobials were considered as possible urinary prophylactic agents in the administrative data analysis even though these agents are not listed in the American Urological Association guidelines. These additional agents included: amoxicillin, ampicillin-sulbactam, carbapenem, 3rd/4th generation cephalosporins, clindamycin, daptomycin, fluconazole, fosfomycin, piperacillin-tazobactam, linezolid, nitrofurantoin, vancomycin

©2018 Khaw C et al. *JAMA Network Open.*
